# Combining low-cost single board computers with open-source software to control noble gas extraction lines

**DOI:** 10.1016/j.mex.2022.101974

**Published:** 2022-12-16

**Authors:** Gary Twinn

**Affiliations:** Department of Earth and Planetary Sciences, Birkbeck, University of London, Malet Street, London WC1E 7HX, UK

**Keywords:** Using low cost single board computers with open source software to control laboratory equipment

## Abstract

Specialised mass spectrometers such as those used in noble gas geochemistry are often controlled by bespoke computer systems that get harder to maintain as, along with their expensive interface cards, they become obsolete. This work explains how bespoke interface cards and obsolete versions of software can be replaced with low-cost single board computers and open source software. Although tailored to an ultra-high vacuum helium extraction line the basic elements and microservices detailed in this paper provide a template for controlling a wider range of instruments.

• Moving from obsolete computer hardware to current hardware.
• Moving from licenced proprietary software to opensource software.
• Automating processes to reduce process time and manual steps.

## Graphical abstract

**Figure:**
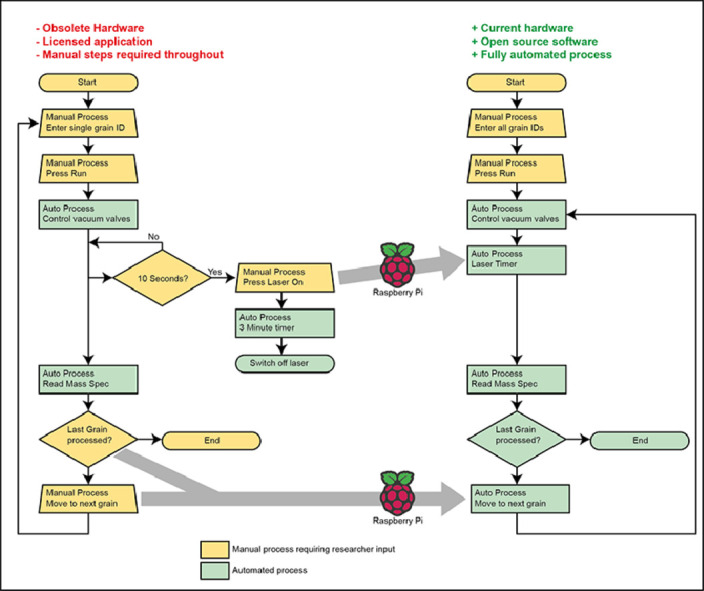

Specifications table

**Table:** 

Subject Area:	Earth and Planetary Science
More specific Subject Area:	(U-Th)/He Geochronology
Method Name:	Using low cost single board computers with open source software to control laboratory equipment
Name and Reference of original method:	Paterson Instruments Helium Extraction Line
Resource availability:	Application code and circuit diagrams can be found at: https://github.com/westerlymerlin/UCL-RPi-PumpReader https://github.com/westerlymerlin/UCL-RPi-XY-Controller https://github.com/westerlymerlin/UCL-RPi-ValveController

## Introduction

Technical debt [3] is a term used to describe the cost of replacing a computer system that is past the end of its supported life. As the time extends beyond the date that support ends, so the cost and complexity of updating the system increases, accumulating technical debt. The current trend in computer science is to try and keep computer systems 'evergreen' by using modern programming languages that are hardware agnostic and running regular incremental upgrades to operating systems. Many examples of technical debt can be found in noble gas laboratories. These typically use self-built or bespoke gas extraction lines to make noble gas (He, Ne, Ar, Kr, and Xe) isotopic and abundance measurements to address a range of geological and environmental problems that include earth formation, origin of the solar system, ocean circulation and dynamics of aquifer systems.

Geologists use helium extraction-purification lines for radiometric age dating. Ages are used to obtain the thermal history of a rock sample to determine the tectonic and environmental processes that have affected that rock and the landscape it formed within. The thermal history can show the timing of tectonic events as well as episodes of burial and erosion giving insights into past climate changes e.g. [22]. The gas extraction line plays a central role in the dating process by measuring and quantifying helium atoms produced by the radioactive decay of their parent atoms of ^238^U, ^235^U, ^232^Th, and ^147^Sm which are present in common rock forming minerals such as apatite, zircon, titanite, and monazite. In detail age determination involves carefully selecting a suitable mineral grain that has been extracted from a rock sample. The grain is then placed in an ultra-high vacuum extraction line where it is heated by a laser to very high temperatures (>900 °C) to liberate the accumulated helium (^4^He), which is then quantified by mass spectrometry against an external ^3^He standard. The final stage of the dating process involves acid digestion of the outgassed grain so that parent isotope concentrations can be determined by isotope dilution techniques on a quadrupole Inductively Coupled Plasma Mass Spectrometer (ICP-MS) instrument.

**Fig. 1:**
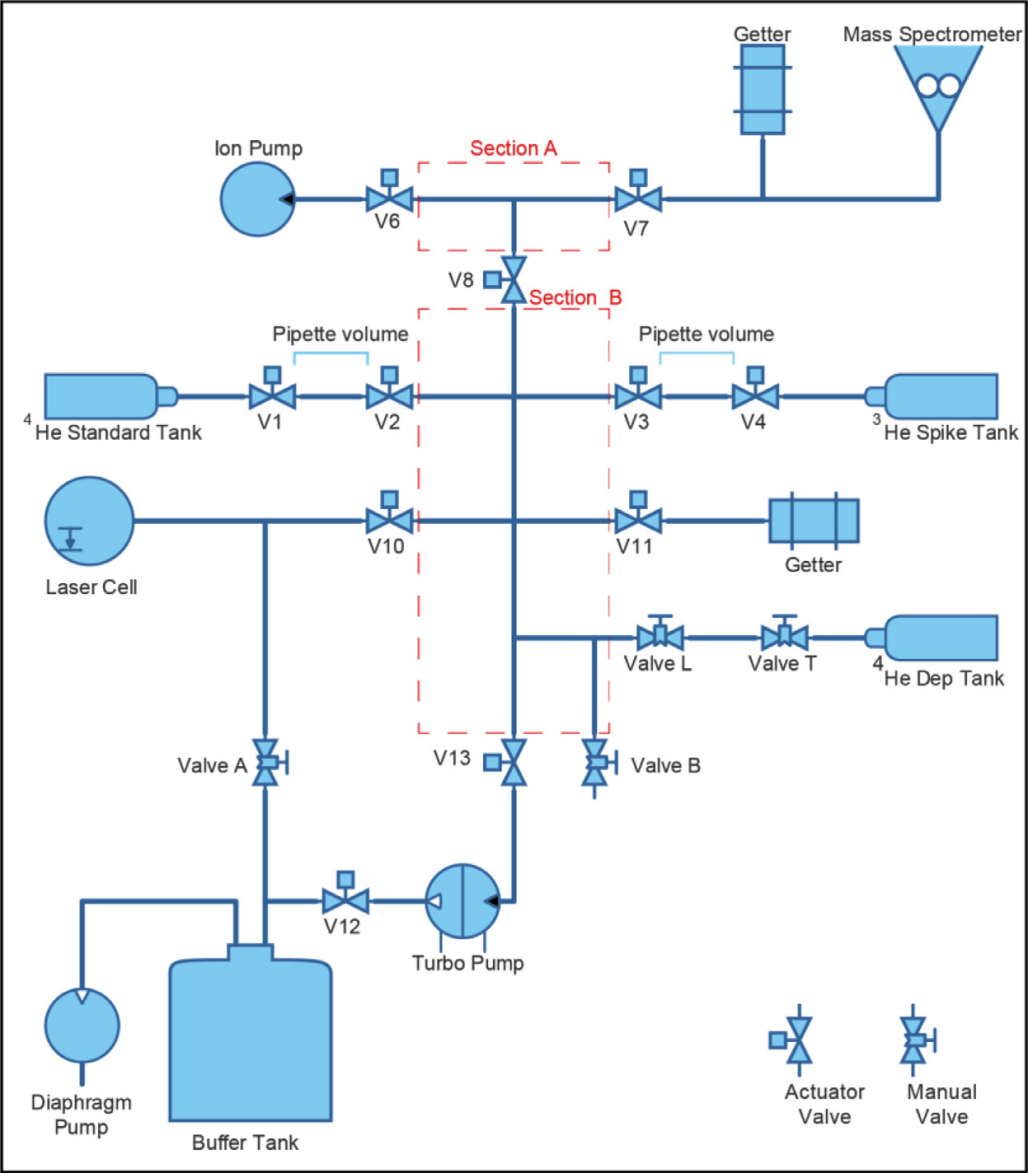
Block Diagram of Helium Extraction Line Valves 1 – 13 are actuator valves controlled by the PC and are operated when outgassing helium from samples, manual valves are only used during sample loading operations. The laser cell contains a copper planchet holding samples beneath a crystal glass to allow laser energy to pass through.

The helium extraction-purification line at the London Geochronology Centre, UCL is a good example of a system that accumulated technical debt. It was commissioned in 2000 and built by Patterson Instruments, a precursor to the more widely used Alphachron™ instrument. It was controlled via a semi-automated LabVIEW [10] process running on a Windows PC. The PC controlled a set of actuator valves on the equipment and read the resulting helium levels from a mass spectrometer (Fig. 1). The process was managed on an outdated version of the LabVIEW control software which only ran on Windows XP. Vendor support for Windows XP ended in April 2009 [8], since then the vendor has not created any security or software bug fixes. The PC hardware running the software was over 19 years old and if the computer had failed it would be unlikely that spares were available. It would have been impossible to replace the computer with a new one as the interface cards would not fit into a new PC. In essence the whole system was running with a growing amount of technical debt. Furthermore, successive modifications to the line had added complexity to the control system, e.g. the upgrade from a resistance furnace to a laser heating system necessitated major changes to the LabView system. It thus became clear that a major overhaul of the control system was needed.

An approach to making feature development less complex is to divide up large monolithic applications into individual microservices. Each microservice operates as separate application and can be independently run and tested as an individual unit. Each microservice will carry out a single task, such as reading from a pressure gauge, and is communicated with via an application programming interface (API) [21]. Rather than replace the existing PC and control cards, install the latest version of LabVIEW to create a new version of the old monolithic solution, a decision was taken at UCL to replace the whole system with a modular solution utilising low cost single-board computers that support General Purpose Input Output (GPIO) and the open-source Python programming language (Python Software [12]). Python is an interpreted language with different versions of the interpreter for different computer types and a common text-based application code. This approach allows the same code to run on different computer types as the base interpreter will convert the application code and tailor it to the commands required for the underlying computer hardware. Python also has an active developer community who develop and share software libraries that carry out lots of useful functions. The goal of this paper is to demonstrate the benefits of replacing all proprietary software and some hardware with open-source alternatives and replacing control cards with low-cost individual single-board computers. The basic elements described in this work therefore have relevance to a wider range of laboratory instruments that experience technical debt.

### Operating process

The purpose of the ultra-high vacuum helium extraction line is to liberate, purify and quantify the amount of ^4^He produced by the radioactive decay of U, Th and Sm present in a sample mineral grain. The approach uses isotope dilution to measure the amount of helium extracted. After heating and liberation of the sample ^4^He a known volume of ^3^He is added via a calibrated pipette (pipette volume = 0.2258 cc ± 0.0012 cc) as a spike to the gas (^4^He) extracted from the grain. Typically, the spike concentration is between 100 and 1000 times higher than the sample ^4^He. At the same time the sample gas is purified using hot and cold SAES getters (SAES Getters S.p.A., [15]) which remove from most of the H_2_O, CO, N_2_, CO_2_, H_2_, and CH_4_. The ^4^He concentration can then be calculated using;

$${}^{4}He=\left({\left({}^{4}He{/}^{3}He\right)}_{sp}-{\left({}^{4}He{/}^{3}He\right)}_{bl}\right){*}^{3}He$$

Where; (^4^He/^3^He)_sp_ and (^4^He/^3^He)_bl_ are the isotopic ratios measured for the sample and blank, respectively, and the amount of 3He is determined from the pipette volume after correction for spike tank depletion.

The operating process prior to upgrade was labour intensive. Firstly, sample grains are placed in 0.5 mm diameter high purity platinum tubes so that laser heating creates a microfurnace. The tubes are then loaded into a copper planchet comprising 55 sample pits. The planchet is positioned into the laser cell (Fig. 1) and the cap is sealed with a copper gasket. The whole system is then pumped down to a vacuum of 1 × 10^-8^ mbar overnight before any samples can be processed. To process a sample the researcher moves the laser assembly above a sample with a joystick and uses a camera to ensure the laser is in the correct place. The researcher then enters the identifier for the sample into the computer system and presses the run button. The software then opens valves 6 and 7 and then close valves 8, 13 and 10. When valve 10 closes the researcher must click a button to activate the LaserVall 1PERACKF21 laser, and trigger a timer to deactivate it after 3 min. The software then opens valve 8 so that the ion pump can purge the sections A and B. After 2 min, valve 8 is closed and valve 4 is opened to fill the pipette with the ^3^He spike. Valve 4 is closed a few seconds later and valves 3 and 10 are opened to allow the measured ^3^He spike along with the ^4^He outgassed from the sample to expands into section B. A minute later valve 6 is closed to shut off the ion pump and valve 8 is opened to allow the gas from section B to flow into section A. The software then takes 20 readings from a Pfeiffer QMS 400 Mass spectrometer at 9 second intervals and writes them away to a file that includes the sample reference entered by the researcher. A separate application written in Java calculates the ^4^He/^3^He ratio at each data point and then performs a regression analysis to calculate the ratio ^4^He/^3^He at the start of the reading and the researcher takes note of that value. The software then opens valve 6 to purge the laser chamber and sections A and B via the ion pump. After 8 min of purging the analytical routines ends. The process takes 18 min and 40 s in total. If the ^4^He/^3^He ratio value is greater than 2.0, the researcher then runs the entire process again to reheat the sample to ensure it is fully outgassed. If the ^4^He/^3^He ratio is less than 1.0 then it is probable that the platinum tube was empty. The researcher then moves the laser to the next sample and repeats the process. At the start of each day a line blank (the same process but with the laser disabled) and a Q-Standard (same process using a known amount of ^4^He released from a pipette) are run to ensure the line is operating correctly. At the end of the day a further line blank and Q-Standard is also run to check that the line is still operating as expected. As a line blank or Q-Standard takes 18 min and 40 s to run, and a sample plus reheat takes 37 min and 20 s at best, a researcher could only run 10 samples in an 8-hour day and therefore a week was required to process a full planchet of 55 samples. A central goal is therefore to replace this manual process with full automation and 24 hour operation, enabling the analytical process to be shortened to just under 2 days, during which the researcher would not need to attend the helium extraction line.

### Solution design

A system analysis of the current functionality of the helium line was undertaken along with a wish-list of new functionalities which were required. The application was divided into a graphical user application that runs on a Windows PC, and several microservices that would run on single board computers with General Purpose Input Output (GPIO) controllers. GPIO consists of a set of software-controlled registers that enable individual control lines to be set to 0 V or 3.3 V which can be used to drive external equipment. The user application was rewritten in Python to run on a PC running the latest version of Microsoft Windows; a Windows PC was specified as it needs to interface with the mass spectrometer application and a pair of cameras all of which only have Windows drivers. The application on the main computer is labelled PyMS (Python Mass Spectroscopy) and includes a graphical user interface as well as databases to record results. The PyMS application is heavily tailored to the cameras, valve solenoids and mass spectrometer for apatite-helium dating at UCL is not easily adaptable to other laboratory equipment, therefore PyMS will not be discussed in detail in this paper.

**Fig. 2:**
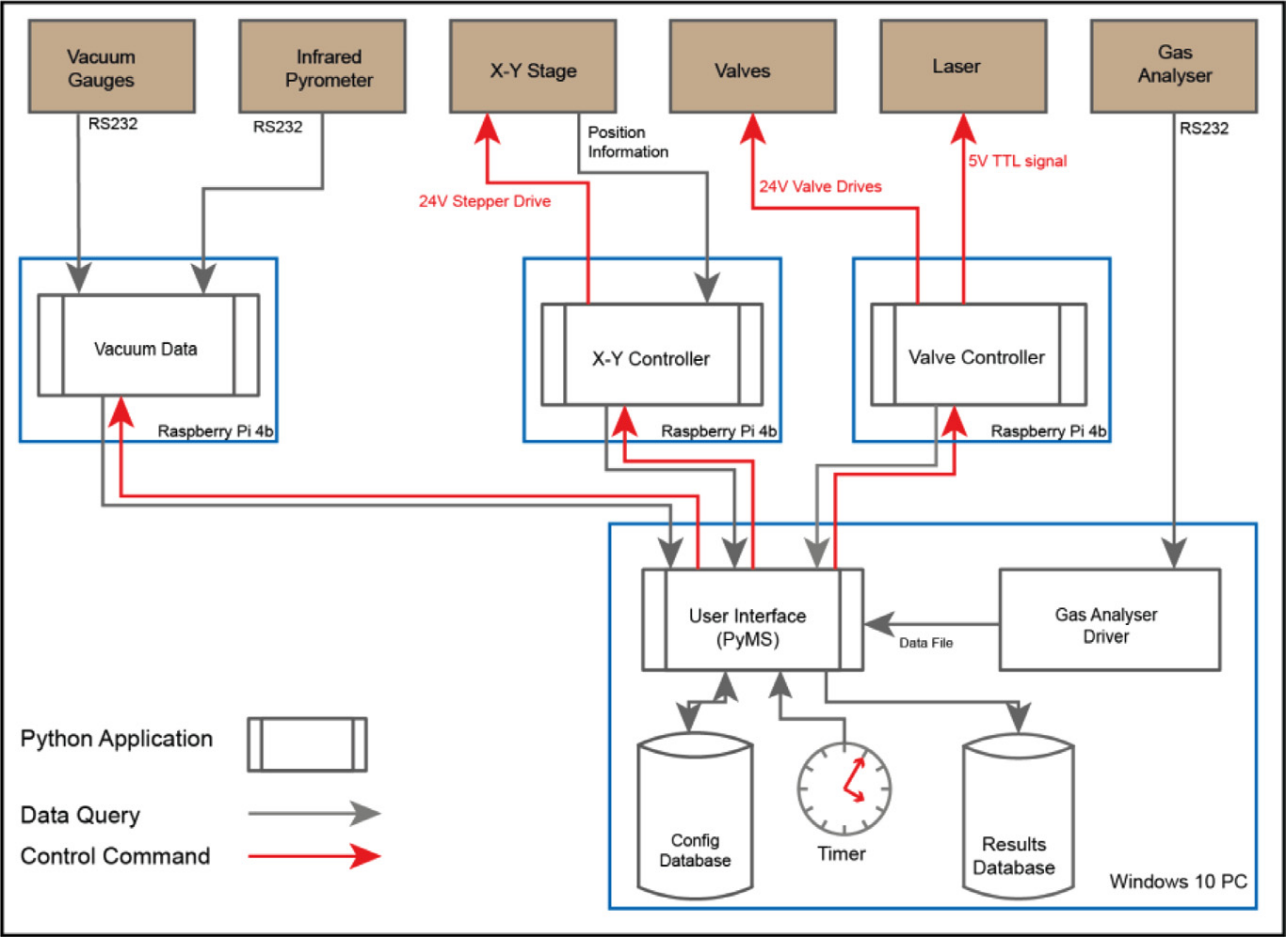
Block Diagram of the solution.

However, three separate tasks were identified that could be split out as microservices and deployed to individual single-board computers to make the control of the line easier to manage (Fig. 2). These are generic services and could easily be tailored to provide low-cost interfaces in other laboratory systems.

1) The control of the vacuum valves and pipettes that control the flow of sample gasses, as well as the pipettes of gas standards is identified as functionality that needs to be replicated on the new system. As the valves are all operated by signals sent to solenoids, the GPIO functionality is used to control the solenoids. The laser that is used to heat the samples is similarly controlled, but by a single +5 V Transistor-Transistor Logic (TTL) signal line and can also be accommodated by the same computer.
2) The control of the X-Y stage was a manual task carried out by the researcher using a joystick to align the sample below the output of the laser via a camera feed to a monitor. As the operation of the helium extraction line is fully automated, the positioning of samples below the laser needs to be controlled by the main computer. The X-Y stage is positioned by a pair of bipolar stepper motors that control the position of the stage via threaded worm screw drives. There is also a position feedback process, so the system knows where the X-Y stage is positioned.
3) For the new solution to be fully automated, there are a number of vacuum gauges on the pumps that need to be read by the main computer, so in the event of a loss of vacuum the system will pause analysing samples. Data for these is available via serial RS232 data connections. On the wish-list is an infrared pyrometer that will be used to read the temperature of a sample as it is heated, this is also read via an RS232 data connection.

Of the available range of single-board computers, the Raspberry Pi 4b [13] was selected due to its low cost, easily controlled GPIO (with libraries for a number of programming languages), built in networking and the availability of add-on interfaces such as analogue to digital converters. The Raspberry Pi 4b has a quad core processor and 4GB of RAM so is more than capable of running a multi-threaded microservice. The software runs on a headless Debian Linux [16] operating system which contains the minimum amount of functionality required to run the application whilst making the lowest demand on the computers resources.

**Fig. 3:**
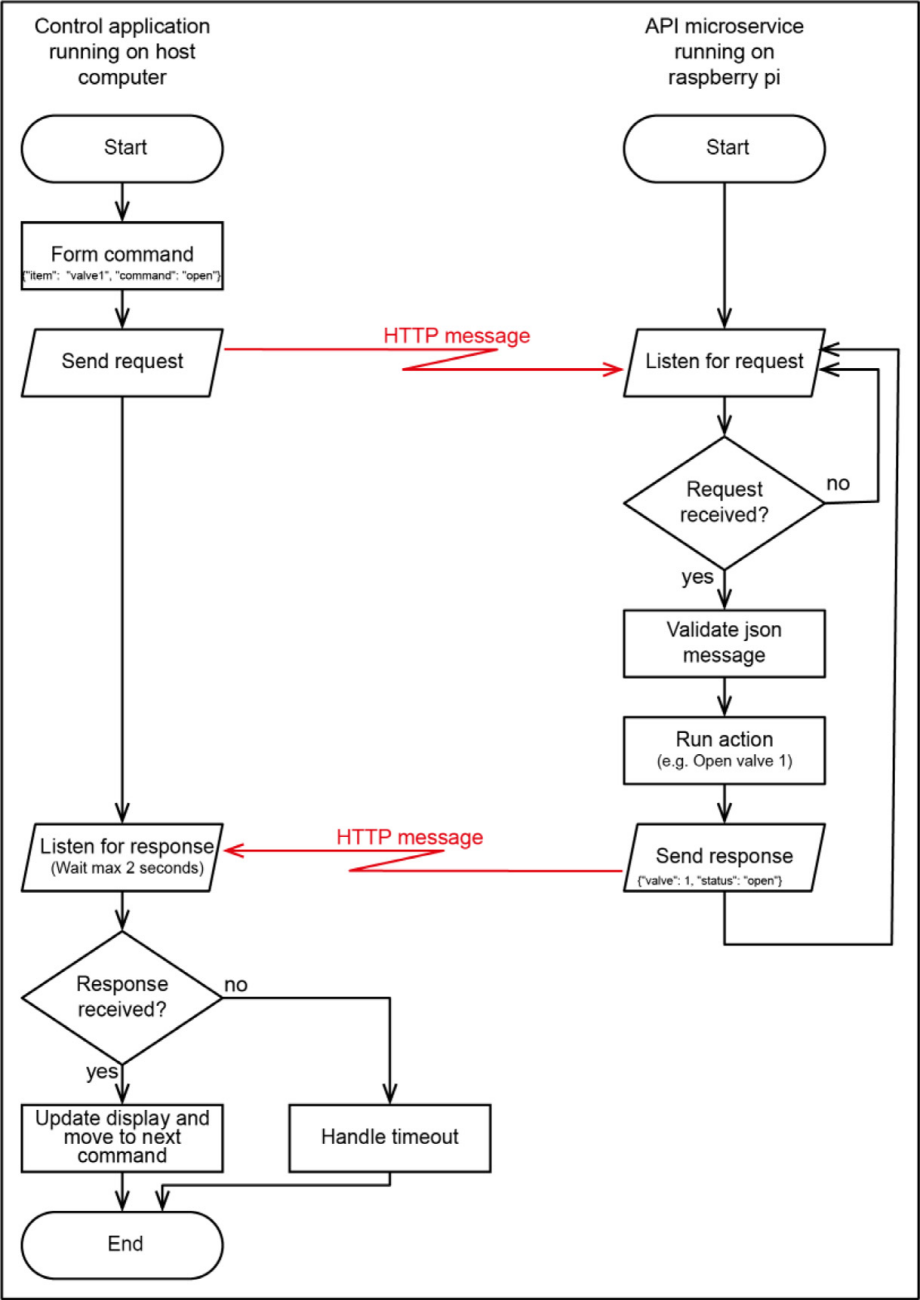
Flow chart of host computer to microservice API communication flow.

Connectivity between the services running on the Raspberry Pis and the main computer on the Helium Line is managed through a 1 Gbit network switch to form a private network. Communication between the main computer and the microservices is through a command / query mechanism via an HTTP network protocol and a REST API [7]. By using a command / query REST protocol, each service was developed separately. As long as the microservices listen for commands on the REST API the whole system is agnostic to programming languages. By using a modular microservice approach an individual microservice can be upgraded without the other services being affected. Messages are sent to and received from the services in a JSON format, an open standard for passing data between systems [2]. Using an HTTP POST request, the command to the microservice is sent in the message header and the response from the requested microservice is returned in the message body (Fig. 3). For the microservices, a common JSON messaging format is used across all three microservices with each command message comprising of two data fields, an “item” which is the piece of equipment that is required to be operated and a “command” which is the action required e.g. {“item”: “valve1”, “command”: “open”} which will instruct the valve controller to cause valve 1 to open. Using this standard messaging format simplifies both the unit testing (testing the functionality of the controlled as a standalone system) of the microservices and writing of the PyMS program that interfaces to them.

The REST API for the microservices is designed around the Flask web application framework [20]. Flask is a Python framework that allows the rapid development and testing of APIs and Web services without the programmer having to code the services that manage web connections. For the microservices, the Flask API connects to a backend Python class to provide the individual functionality for the service. There is also a basic set of web pages generated by Flask to show a read-only status page for the API as well as access to the application log files, system log and webserver error logs for troubleshooting issues. Although the Flask framework contains a built in web server for use during application development, it is not robust enough for hosting a production service [14], so on the Raspberry Pi 4b, a Gunicorn webserver [1] and Nginx reverse proxy [18] are implemented. Gunicorn allows for multiple worker threads and has a better security model than Flask, while Nginx handles the network connections and ensures only valid HTTP requests are routed to the Python code.

All of the python application development work is carried out using the JetBrains PyCharm IDE [6] and unit testing is carried out via the Postman REST testing application [11]. Version control of the developed software is managed by Github.com.

### Valve control

The vacuum valves that control the flow of gasses through the helium line are operated by 24 V solenoids. Each solenoid requires a 100 mA current to open the valve and removing the current allows the valve to close against a spring. The Raspberry Pi GPIO has a 3.3 V signal voltage and can supply a maximum current of 16 mA [13] which means a driver electronic circuit is required to step up the voltage and current for the solenoids.

**Fig. 4:**
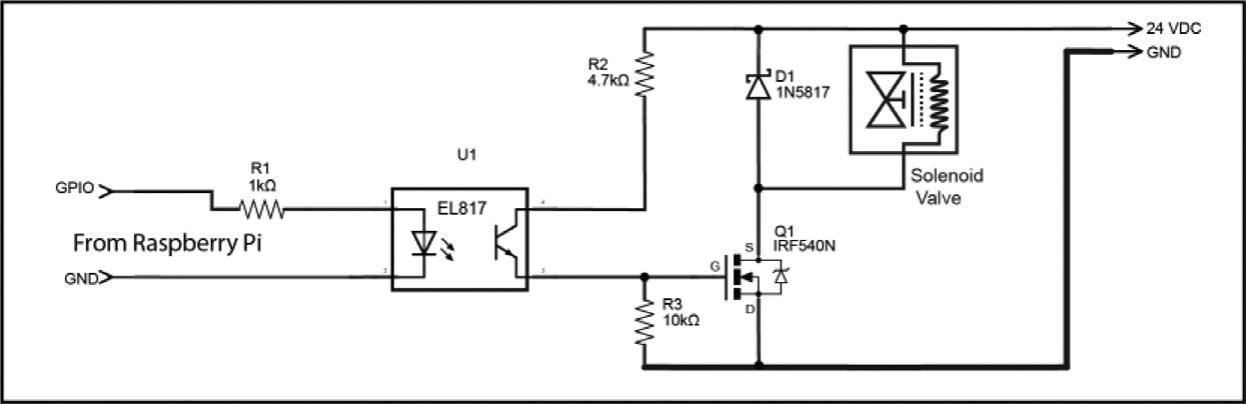
Valve Driver Schematic.

To provide the necessary power to drive the solenoids, a driver circuit board has been developed that uses one Metal Oxide Field Effect Transistors (MOSFET) per valve (Fig. 4**. Q1**). MOSFETs have a very low forward voltage once switched on and have a high-power capacity [4]. They act as an electronic switch with a small voltage and current on the gate allowing a larger current to flow through from the source to the drain. MOSFETs were chosen over using relays in this instance as MOSFETs are considerably cheaper than relays and the raspberry PI GPIO output current is insufficient to drive a relay directly without a transistor driver circuit.. During the process, a maximum of 10 valves are actuated simultaneously, so the power supply needs to supply a minimum of 1.0 A continuously. To provide the required level of reliability, a 24 V power supply with a continuous rating of 3.0 A is specified to operate the valves. Whilst the GPIO output of the computer could be connected directly to control the MOSFET, it could be damaged by a circuit fault or spike on the 24 V power line. To prevent possible damage to the computer an opto-isolator was included in the driver circuit (Fig. 4**. U1**). An opto-isolator consists of an LED that is coupled via a tunnel to a phototransistor, when a signal voltage is applied to the LED it lights and the phototransistor switches on. The air gap means there is no electrical connection between the high voltage and low voltage sides of the circuit. As the valves are operated by inductive solenoids, when the power is removed from the solenoids the collapse of the magnetic field induces voltage spikes into the circuit, so a Schottky diode (Fig. 4**. D1**) was added to protect the MOSFET and opto-isolator.

The GPIO channels that operate the valves are controlled through a standard RPI.GPIO Python library. The valve controller microservice contains a mapping variable that links valve numbers (as specified in the PyMS application) to the Raspberry Pi 4b GPIO channels. The microservice also uses a GPIO channel to illuminate an LED once the application has loaded and is ready to receive commands. The Python code and a readme file containing circuit diagrams can be downloaded from https://github.com/westerlymerlin/UCL-RPi-ValveController.

The LaserVall 1PERACKF21 Nd:YAG 808/1064 nm laser requires *a* + 5v TTL output to enable the beam, however the Raspberry GPIO consists of a 3.3v Complementary Metal Oxide Semiconductor (CMOS) integrated circuit. In order to setup-up the signal voltage a SN74HC125NE4 buffer integrated circuit (Texas [19]) was used to convert the CMOS signal to the TTL for the Laser control.

Messages arrive at the microservice via HTTP messages with a JSON payload. The Flask framework receives the HTTP POST message, decodes the JSON and checks it for validity. If the message is valid it routes the message to the parsecontrol function within the valvecontroller.py module, determining which valve needs to be operated and identifying the operation. The Flask framework returns a JSON message that contains each valve name and its status (open or closed) (Fig. 3). The status message can also be retrieved by the command {"item": "getstatus", "command": "c"}.

There are two calibrated pipettes within the helium line, each with a valve at each end. One end is connected to a gas standard tank containing isotopically pure helium and the other to the main body of the instrument (Fig. 1). At no point should both the inlet and exhaust valves of a pipette be open at the same time, so logic was required to prevent this. The Python mapping variable that describes the valves and GPIO connections also makes provision for an excluded combination, so if an inlet valve for a pipette is open then the code will not allow the exhaust valve to be opened. The code also allows for an emergency stop command that closes all valves simultaneously.

### X-Y controller

**Fig. 5:**
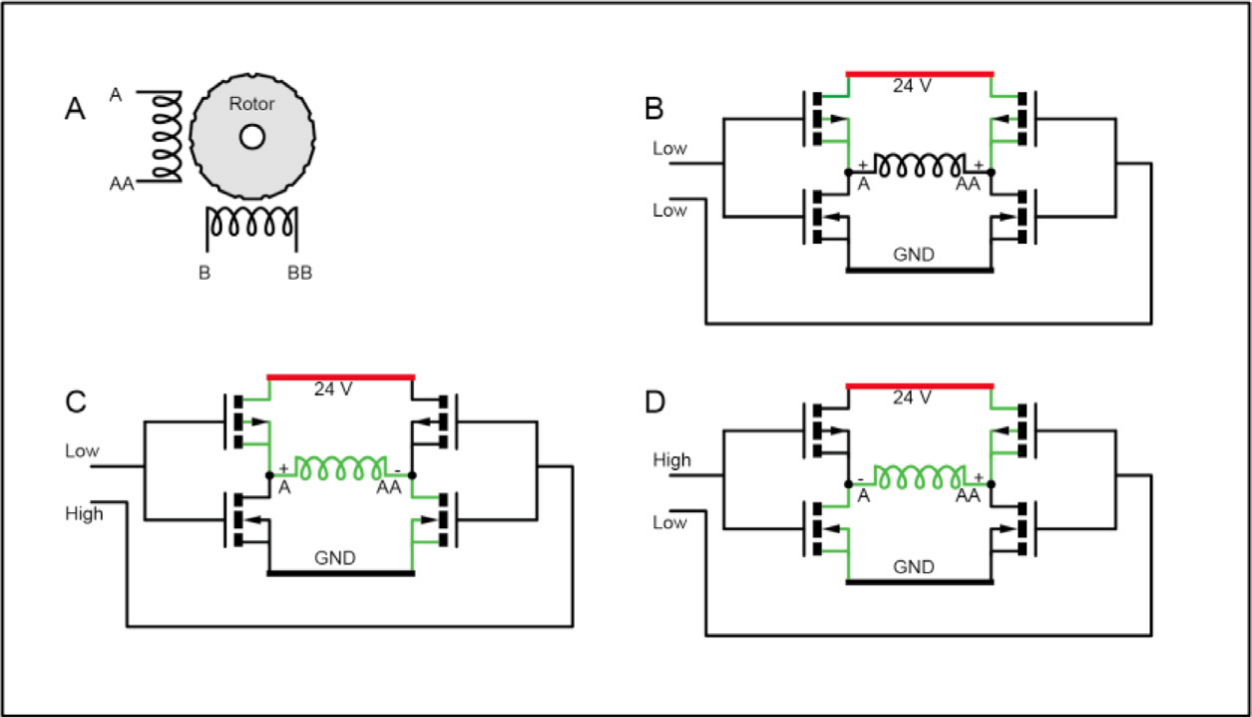
A) Bipolar Stepper Motor. B) H-Bridge stop position. C) H-Bridge energised *A*+. D) H-Bridge energised AA+.

A copper planchet with 49 × 1 mm sample pits drilled in a 7 × 7 square is located in the laser cell beneath a crystal window. The positioning system for heating samples involves a laser mounted on an X-Y stage driven by a pair of 24 V bipolar stepper motors connected to worm gears. Bipolar stepper motors have a pair of coils positioned around a rotor containing permanent magnets (Fig. 5**A**). By energising the coils, the fixed magnets will either be attracted or repelled by the magnetic field in the coils and the stepper will rotate by a fixed increment. As each connection on a coil needs to be either connected to the + or – supply, an H-Bridge circuit is utilised to power each coil (Fig. 5**B - D**) [5]. An H-Bridge works by having two pairs of MOSFET transistors, each pair having an N channel connected to ground and a P channel connected to the supply rail and sharing an input line connected to both gates. If no signal is applied to either input, both ends of the motor coil will be connected to the supply rail and the coil will not be energised (Fig. 5**B**). If a signal is applied to one of the inputs, that set of MOSFETs will power on the N channel transistor and power off the P channel transistor connecting that coil terminal to the ground power rail, energising the coil to attract the magnets one way. Connecting the coil terminals the other way around reverses the magnetic field in the coils and repels the magnets.

**Fig. 6:**
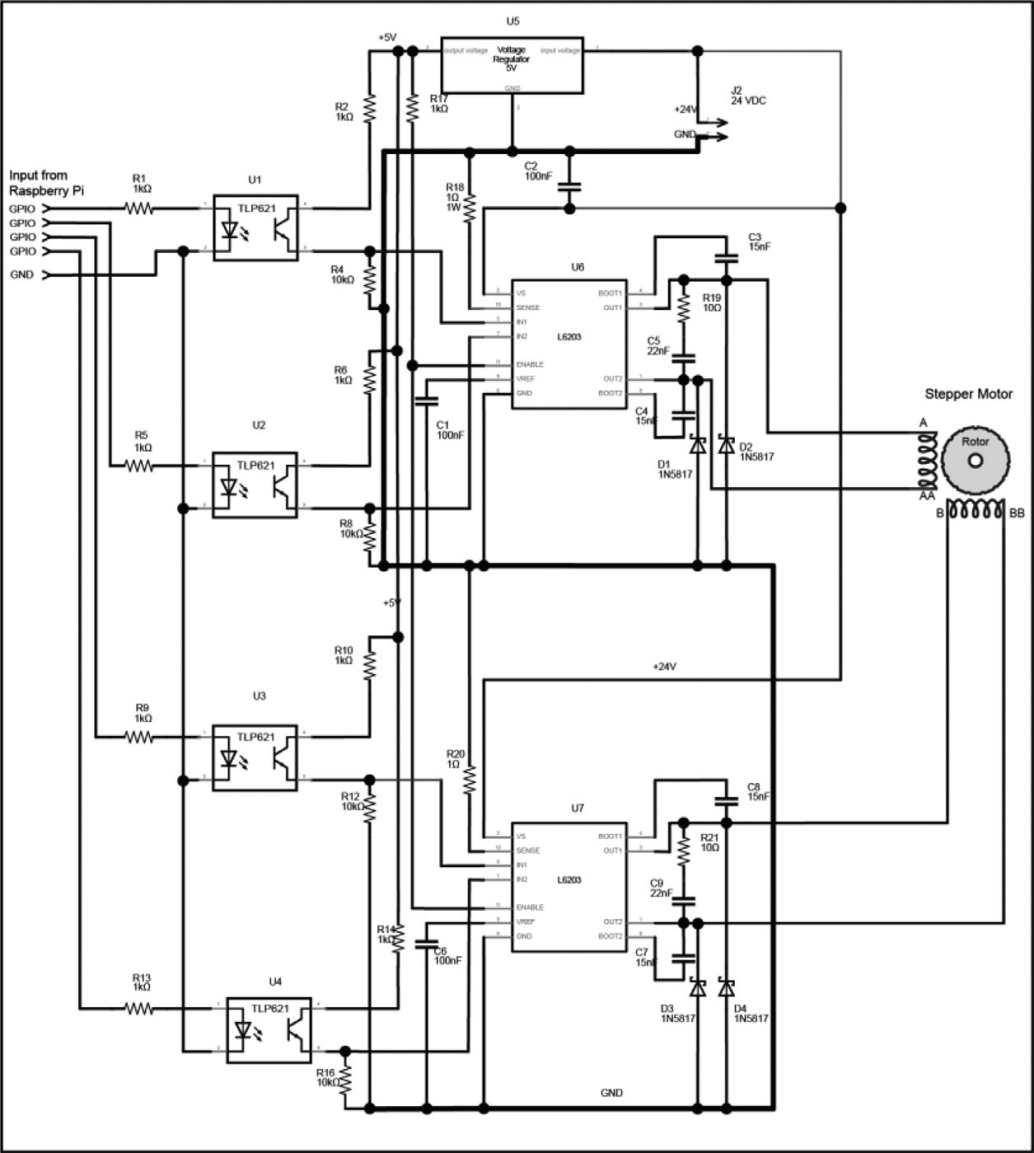
Stepper Motor Driver Schematic adapted from [17].

Rather than use discrete MOSFETs which would complicate the circuit, an L6203 integrated driver circuit that contains the H-Bridge as well as additional thermal protection and driver circuits [17] is used to drive the motor coils. Two integrated circuits per motor are needed, one for each motor coil, and the inputs are connected to opto-isolators to protect the Raspberry Pi (Fig. 6).

In order to move the X-Y stage to a sample position, a feedback mechanism is required to inform the system as to the location of the laser in relation to the location of the sample to be processed. Three approaches were considered: A first approach was to count the steps taken by the motor from a datum. Each step is 1.8° which equates to around 10 µm of travel on our stage, however this step size is subject to some noise which accumulates with distance travelled. A second approach uses one of the cameras mounted on the laser assembly and artificial-intelligence image processing to calculate where the laser is located. This method was discounted as being too complex. Ultimately, we chose a third approach which uses a pair of linear positioning potentiometers, one on each axis and an analogue-to-digital convertor interface attached to the Raspberry Pi to feed back the actual position of the X-Y stage. The potentiometers have a 50 mm travel and by attaching one end of the potentiometer to a 5 V power rail and the other to ground, the wiper registers a voltage difference of 100 mV per mm of travel. The analogue-to-digital convertor was configured to have a resolution of 4096 bits so the minimum voltage difference it could detect is 1.2 mV which equates to 12 µm which is close to the minimum travel in 1 step. As each sample location is 1 mm in diameter and the laser beam width is 1.2 mm, having a location to within 12 µm is accurate enough to ensure a good transfer of heating energy from the laser. The microservice does not store details of locations but accepts requests to move to a voltage level, so an application calling the microservice would issue a command {"item": "xmoveto", "command": 0.711} and the X-Y controller would start generating step signals to the stepper motor until the x potentiometer gave a reading of 0.711 V.

The X-Y controller Python microservice as designed to be multi-threaded so once a command is issued the application generates a new thread to deal with that command and immediately returns to listening for additional requests. Once the command is completed, the thread is deleted so the Raspberry Pi does not run out of memory or processor resources. When the GPIO signal applies power to the stepper motor coils, there must be a small delay (50 ms) so the magnets on the rotor can move before the next step is signalled. As the location voltage approaches the desired voltage the delay is increased and an additional reading of the analogue-to-digital convertor is instigated. Once the location voltage has passed the desired position voltage, the stepper motor is moved back one step. The Python code as well as a readme file containing circuit diagrams can be downloaded from https://github.com/westerlymerlin/UCL-RPi-XY-Controller

### Vacuum gauge reader

The helium line contains two Pfeiffer vacuum gauges, a Gamma Vacuum ion pump and a Micro Epsilon Infrared Pyrometer. To provide fault detection to the new PyMS system the vacuum pressures of the turbo pump and ion pump need to be monitored and if either pressure rises above a threshold the PyMS system will stop processing samples. The pyrometer reading is required to understand what temperature the samples are heated to.

Both the Pfeiffer and Gamma devices provide a computer interface via an RS232 serial interface. Pressure and other information on the status of the pumps is available via commands sent through the RS232 interface, when the interface receives a command it then returns the data requested.

**Fig. 7:**
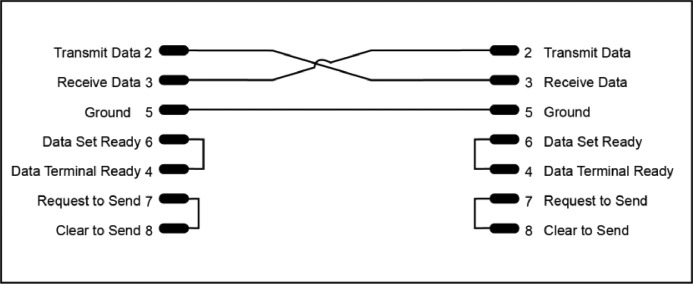
RS232 null modem cable wiring diagram.

The Raspberry Pi has a single serial interface available on GPIO connections 14 and 15, however the requirement for the gauge reader was a minimum of 3 serial connections. The Raspberry Pi 4b has 4 USB connections, there is an inexpensive USB to serial adaptor that utilises the CH340 chipset (Nanjing Qinheng Microelectronics Co., [9]). The USB Serial connections are made available to the Linux operating system under the device identifiers /dev/ttyUSB0 to /dev/ttyUSB3. To connect the CH340 RS232 connector to the RS232 connectors on the vacuum gauges a null modem is required that links the Transmit Data (TD) connection on the computer with the Receive Data (RD) connection on the gauge and the RD connection on the computer to the TD on the gauge (Fig. 7).

The Python code in the microservice that reads the data from the instruments is set to read from each instrument every 4 s and runs as a multithreaded application. The Pfeiffer gauges require two data packets to be transmitted, one which defines the type of data required (in this case the pressure) and a second command that is a read instruction, the gauge then returns a string with the pressure to the RS332 port where it is read by the Raspberry Pi. The pyrometer and ion pump only require a single read command to retrieve data. Once the data is retrieved from each device it is made available via the REST API and is read via the “getpressures” or “gettemperatures” command string. The Python code can be downloaded from https://github.com/westerlymerlin/UCL-RPi-PumpReader.

## Competing interests

The authors declare that they have no known competing financial interests or personal relationships that could have appeared to influence the work reported in this paper.

## Data Availability

No data was used for the research described in the article.
